# Can We Predict Imbalance in Patients? Analysis of the CDC National Health and Nutrition Examination Survey

**DOI:** 10.3390/jcm12051943

**Published:** 2023-03-01

**Authors:** Bassel G. Diebo, Sarah G. Stroud, Neil V. Shah, James Messina, James M. Hong, Daniel Alsoof, Kashif Ansari, Renaud Lafage, Peter G. Passias, Virginie Lafage, Frank J. Schwab, Carl B. Paulino, Roy Aaron, Alan H. Daniels

**Affiliations:** 1 Department of Orthopedics, Warren Alpert Medical School, Brown University, Providence, RI 02903, USA; 2 Department of Orthopaedic Surgery and Rehabilitation Medicine, The State University of New York Downstate Health Sciences University, New York, NY 11203, USA; 3 Department of Orthopedics, University of Connecticut, Farmington, CT 06032, USA; 4 Department of Orthopaedic Surgery, Hospital for Special Surgery, New York, NY 10021, USA; 5 Department of Orthopedic Surgery, NYU Langone Orthopedic Hospital, New York, NY 10010, USA; 6 Department of Orthopaedic Surgery, Lenox Hill Hospital, Northwell Health, New York, NY 10075, USA; 7 Department of Orthopaedic Surgery, New York-Presbyterian Brooklyn Methodist Hospital, New York, NY 11215, USA

**Keywords:** imbalance, fall risk, frailty, functional assessment, nutritional assessment, CDC NHANES

## Abstract

Understanding global body balance can optimize the postoperative course for patients undergoing spinal or lower limb surgical realignment. This observational cohort study aimed to characterize patients with reported imbalance and identify predictors. The CDC establishes a representative sample annually via the NHANES. All participants who said “yes” (Imbalanced) or “no” (Balanced) to the following question were identified from 1999–2004: “During the past 12 months, have you had dizziness, difficulty with balance or difficulty with falling?” Univariate analyses compared Imbalanced versus Balanced subjects and binary logistic regression modeling predicted for Imbalance. Of 9964 patients, imbalanced (26.5%) were older (65.4 vs. 60.6 years), with more females (60% vs. 48%). Imbalanced subjects reported higher rates of comorbidities, including osteoporosis (14.4% vs. 6.6%), arthritis (51.6% vs. 31.9%), and low back pain (54.4% vs 32.7%). Imbalanced patients had more difficulty with activities, including climbing 10 steps (43.8% vs. 21%) and stooping/crouching/kneeling (74.3% vs. 44.7%), and they needed greater time to walk 20 feet (9.5 vs. 7.1 s). Imbalanced subjects had significantly lower caloric and dietary intake. Regression revealed that difficulties using fingers to grasp small objects (OR: 1.73), female gender (OR: 1.43), difficulties with prolonged standing (OR: 1.29), difficulties stooping/crouching/kneeling (OR: 1.28), and increased time to walk 20 feet (OR: 1.06) were independent predictors of Imbalance (all *p* < 0.05). Imbalanced patients were found to have identifiable comorbidities and were detectable using simple functional assessments. Structured tests that assess dynamic functional status may be useful for preoperative optimization and risk-stratification for patients undergoing spinal or lower limb surgical realignment.

## 1. Introduction

Maintaining total body balance is a complex process with static and dynamic components that relies upon visual, vestibular, and proprioceptive information [1,2]. These inputs are predominantly analyzed by cerebellar and brainstem centers, which compare body position against a movement plan provided by the cerebral cortex and respond by coordinating appropriate changes in eye movements and tone of the axial musculature [3,4]. Dysfunction in any of the above sensory systems or the central controllers, as well as failure of motor output or muscle weakness may result in an inability to maintain balance.

Postural stability has been reported to decrease as a person ages, with resultant increases in postural sway [5,6]. Elderly persons may have dysfunction in any of the systems described above; for example, they may display diminished visual acuity and increased muscular weakness compared to younger adults [7,8]. Additionally, patients with benign paroxysmal positional vertigo experience difficulty sustaining a quality gait and often suffer from impaired walking stability [9]. Postural stability is known to be dependent on the information retrieved by the visual, vestibular, and proprioceptive systems, and thus, damage to any of these systems can have detrimental effects on one’s postural stability. Accordingly, there has been shown to be a positive relationship between hearing loss and postural instability, a relationship that is also apparent for amblyopia patients and those who experience cataractous blurs in their vision [10,11,12]. Postural instability can also be a side effect of several medications, including benzodiazepines, which have caused falls and hip fractures after its usage in elderly patients [13]. Orthostatic hypotension is one of the most common causes of temporary postural instability and subsequent falls, which is why loop diuretics, anti-hypertensives, antipsychotics, and tricyclic antidepressants all increase the risks of postural instability [14]. Conversely, some medications that have been prescribed for postural stability complications include methylphenidate, which is associated with preventing gait freezing, and donepezil, which has benefits regarding a reduced risk of falling [15]. 

Regardless of the underlying cause, imbalance of any kind predisposes to falls, which have been identified as the leading cause of accidental deaths among adults in the United States older than 65 years old [16]. Falls are also associated with high morbidity, with approximately 25% of patients experiencing a moderate to severe injury [17]. Falls are so costly to quality of life among the elderly that the American Academy of Orthopaedic Surgeons (AAOS), in conjunction with the Orthopaedic Trauma Association (OTA), elevated fall prevention to the forefront of its 2018 Public Service Advertising (PSA) campaign, which emphasized taking any step possible to reduce fall risk [18,19]. Balance impairment and muscle weakness have been identified among the strongest modifiable risk factors for falls in elderly patients, such that the American Geriatrics Society (AGS) recommends screening for a history of imbalance in patients over sixty-five [17].

Though the risk factors for body imbalance or sustaining a fall have been proposed in the literature, there is a paucity of evidence supporting those predictors. Most data is derived from single centers, or a single specialty, with little known about prediction of imbalanced patients on the national level [20,21,22,23]. Using a nationally representative sample, this study examined a population of patients with imbalance to identify demographics, comorbidities, laboratory values, and functional assessments associated with the condition. 

## 2. Materials and Methods

The Centers for Disease Control and Prevention (CDC) establishes a representative sample annually via the National Health and Nutrition Examination Survey (NHANES). All the counties in the United States are divided into 15 groups based on their characteristics. One is selected from each group and together they form the 15 counties in the NHANES survey for the year. Within each of the 15 NHANES counties, smaller groups (such as neighborhoods) are formed, and between 20 and 24 of these small groups are selected. All the houses or apartments within those selected small groups are identified, and a sample of about 30 households are chosen within each group. NHANES will contact the selected households and ask a short set of questions (age, race, and gender). A computer process randomly selects some, all, or none of the household members. In total, NHANES annually includes approximately 5000 individuals to assess the health and nutritional status of adults and children through a combination of interviews and physical exams. This ongoing data collection has helped identify health problems and risk factors in the U.S. population over time, assisting in the development and improvement of various education and health programs across the country [24]. In a retrospective review of this database, NHANES was searched from 1999–2004 (*n* = 31,126), the years for which imbalance data was collected by the CDC, to identify all patients that responded to the question: “During the past 12 months, have you had dizziness, difficulty with balance, or difficulty with falling?” Patients who answered “yes” were included in the Imbalanced group, while patients who answered “no” were labeled Balanced. 

In univariate analyses, Imbalanced patients were compared against Balanced patients with respect to demographics, presence of comorbidities, nutritional parameters, physical assessments and performance measures, and laboratory tests. The Kolmogorov–Smirnov test was utilized to test normal distribution of the data. Mean and standard deviations, medians, and interquartile ranges (IQR) were reported appropriately and compared using student’s *t*-tests and Mann–Whitney U tests, respectively. Factors that had a statistical difference of *p* < 0.1 in univariate analyses were used to structure a binary logistic regression model. The model was utilized to identify independent predictors of Imbalanced patients. Each analysis only included entries with valid data to account for the possible presence of missing data. The threshold for statistical significance was set at *p* < 0.05, and odds ratio (OR) and confidence intervals (CI) were calculated for each factor. 

## 3. Results

### 3.1. Demographics and Comorbidities 

Of the 9964 patients who provided a valid response to the NHANES Balance question, 2638 (26.5%) had imbalance and 7326 (73.5%) reported no imbalance. Imbalanced patients had a higher mean age when compared to Balanced patients (65.4 vs. 60.6 years, *p* < 0.05) and were more often female (60% vs. 48%, *p* < 0.05). Race distribution was comparable between groups. The Imbalanced group had a higher prevalence of osteoporosis (14.4% vs. 6.6%), arthritis (51.6% vs. 31.9%), low back (54.4% vs. 32.7%) and neck pain (10.8% vs. 4.3%), and depression or anxiety (1.5% vs. 0.6%); all comparisons had *p* < 0.001 (Table 1). Other comorbidities were evaluated but not reported due to non-significant *p*-values. 

### 3.2. Laboratory Values

Laboratory values were comparable or had clinically irrelevant differences (Table 2). Results were comprised of standard comprehensive metabolic panels, and both Balanced and Imbalanced groups fell within normal acceptable ranges, as defined by AMA guidelines [25]. However, the Imbalanced group had significantly lower dietary calorie intake and lower intake of dietary fiber, protein, vitamins B1, B2, B3, B6, D, and C, calcium, phosphorus, Alpha carotene, and Beta carotene (all *p* < 0.05; as shown in Table 3) [26].

### 3.3. Functional Assessments

Imbalanced patients more often reported difficulty walking up 10 steps (43.8% vs. 21.0%), assuming stooping, crouching, or kneeling positions (74.3% vs. 45.7%), standing up from an armless chair (48.6% vs. 21.0%), and standing up on their own (4.0% vs. 0.9%, all *p* < 0.001) (Table 4). Imbalanced patients also needed a longer time to walk 8 feet (3.3 vs. 2.7 s) and 20 feet (9.5 vs. 7.1 s, all *p* < 0.001).

### 3.4. Independent Predictors of Imbalance

Logistic regression revealed that difficulty using fingers to grasp small objects (OR: 1.73; 95% CI: 1.34–2.24, *p* < 0.001), female sex (OR: 1.43; 95% CI: 1.12–1.83, *p* = 0.004), difficulty standing for long periods of time (OR: 1.29; 95% CI: 1.11–1.51, *p* = 0.001), difficulty stooping, crouching, or kneeling (OR: 1.28; 95% CI: 1.09–1.51, *p* = 0.002), and increased time to walk 20 feet (OR: 1.06; 95% CI: 1.01–1.11, *p* = 0.009) were all significant independent predictors of reporting imbalance.

## 4. Discussion

Given the high morbidity and mortality of falls in those over age 65, fall prevention has entered the national spotlight [18,19]. Identification of those at risk is the first step in creating a comprehensive fall prevention strategy. In the present study, among 9964 responders to the National Health and Nutrition Examination Survey about imbalance during a 15-year period, about one in every four self-reported imbalance, a proxy for fall risk (26.5%). Imbalanced patients were more likely to be older females and had a higher prevalence of osteoporosis and depression/anxiety. These patients also performed worse on select functional tests, such as standing up from an armless chair, and had less caloric intake and dietary intake of essential vitamins and nutrients when compared to patients in the Balanced cohort. While data about the prevalence of imbalance in patients with Parkinson’s and Alzheimer’s disease exist, to our knowledge this is the first study to report the prevalence of imbalance of any type in a nationally represented adult population [27,28].

Though data specifically regarding imbalance are scarce, prevalence of imbalanced patients in our study is in agreement with a previously published study by Ganz et al. [20] in 2007. The finding that patients in the Imbalanced group were more likely to be female is also in agreement with the literature [29]; despite this, it is difficult to identify the mechanisms underlying the disparity between sexes. In fact, older men have previously been shown to be less stable when standing with feet together as compared to older women [17,30]. However, a second study found that controlling for subject height was shown to mostly eliminate a similar effect of sex on balance [31]. Meanwhile, older women have been reported to be more likely to exhibit dizziness and non-neurologic gait disorders, both possible causes of imbalance, when compared to older men [32,33]. The prevalence of sarcopenia, yet another potential cause of imbalance, has not been found to vary between men and women [34]. However, the female sex has been reported to be a risk factor for malnutrition among the elderly [35].

The relationship between nourishment and falls has been studied previously. Gray-Donald et al. [36] found that the number of falls was decreased in frail elderly patients after nutritional supplementation. This is concordant with the results of the present study, showing an association between imbalance and decreased caloric intake and decreased dietary intake of essential vitamins and nutrients. Relative undernourishment in the Imbalanced group compared to the Balanced group may have bidirectional effects on the development of postural instability. For example, subacute combined degeneration due to vitamin B12 deficiency may cause imbalance due to impairment of proprioceptive inputs and motor outputs, and the resultant functional decline may exacerbate malnutrition by preventing patients from completing their activities of daily living. Whether cause or effect, however, our results suggest that the presence of decreased caloric intake or decreased intake of vitamins and nutrients in an older patient should trigger screening for imbalance.

In order to target such screening, it is useful to consider the fact that among the functional assessments performed, several were more predictive of imbalance. Specifically, grasping of small objects, difficulty standing for long periods of time, difficulty assuming the postures of stooping, crouching, or kneeling, and increased time to walk 20 feet were associated with the presence of imbalance. Hand grip strength has previously been reported to be predictive of falls in older adults and associated with better performance on force platform balance tests [31,37]. Among the clinical assessments of gait, strength, and posture suggested for use by the American Geriatrics Society (the Get Up and Go Test, Timed Up and Go Test, Tinetti Performance-Oriented Mobility Assessment (POMA), and Berg Balance Scale), none contain all of the assessments found to be predictive of Imbalance in the present study [38,39,40,41]. However, slight modifications could be made to the Tinetti or Berg protocols to include hand grip, crouching or stooping postures, and a longer walking distance, in order to encompass each of the predictive features found in the present study. Such tests are useful because they do not require specialized equipment, such as a pressure-sensing platform, and can be performed during a routine office visit by providers of any specialty once a risk factor for imbalance becomes apparent. Screening for imbalance via the functional assessments identified in this study may provide a simplified and standardized method by which geriatric patients managed by multiple doctors across multiple disciplines may be assessed.

Future studies may endeavor to compare a composite assessment of hand grip, prolonged standing, walking 20 feet, and assuming crouching, stooping, or kneeling positions against the Berg Balance Scale, the POMA, or perhaps structure a systematic test, such as the Dubousset function test (DFT). The DFT is a four-component test that aims to evaluate functionality, muscle integrity, and overall balance during motion that can be easily performed in less than a minute with minimal equipment required in healthy asymptomatic subjects [42]. Recent investigation in a population of patients with spinal deformity and lumbar degenerative disease has shown moderate to strong correlations between the DFT components and patient-reported outcome measures, including the Oswestry Disability Index (ODI), the Neck Disability Index (NDI), and the Montréal Cognitive Assessment (MoCA), potentially representing a powerful tool to assess balance, among other factors, in spine patients [43]. In addition, future studies may also analyze the association between imbalance and malnourishment as assessed by an established tool, such as the Mini Nutritional Assessment (MNA), versus dietary intake, as done in the present study [44]. Future efforts should also seek to better define the heterogeneous population of patients who suffer from imbalance, particularly with respect to how presentation and performance on functional clinical assessments may vary with the underlying cause.

This study was not without limitations. While we found the Imbalanced group to have a higher prevalence of osteoporosis, arthritis, low back and neck pain, and anxiety or depression, however, the effects of these conditions on presentation were not assessed. Although covering an extensive length of time, the NHANES data does not have the specificity necessary to conduct a rigorous analysis on all imbalance determinants. For example, several potentially relevant factors, including sarcopenia, physical activity, physical training, medications, disorienting sensory conditions, frailty, etc., were not included as predictors in the database. This limitation is due in part to both the retrospective and self-reported nature of the NHANES data, as well the absence of several known causes of imbalance, such as vestibulopathies or Parkinson’s disease, from comparative analysis. In addition, individual underlying conditions may have impacted the ability of patients to attempt the dynamic functional assessments described above or may independently affect performance. Furthermore, while BMI and height could be significant factors regarding imbalance, these aspects were not present in the dataset analyzed. Studies are underway for more granular and prospectively collected data on functional assessments and their ability to predict imbalanced patients. Future studies should include the additional factors noted above and comprehensively investigate whether those comorbidities present any notable relationships with postural stability.

## 5. Conclusions

To our knowledge, this is among the first studies to establish a prevalence rate of imbalance in a nationally representative population of adults in the United States. These results provide evidence on predictors that may potentially aid in identifying imbalanced patients, which may subsequently reduce or prevent falls. The nature of these results also illustrates the glaring need for a simple, universal, and multidisciplinary functional assessment test that can identify at-risk patients across multiple specialties and patient care settings. Functional testing may be a mechanism by which orthopaedic surgeons can play their part in the AAOS’ public safety campaign by identifying dynamic functional impairment in patients to mitigate fall risk.

## Figures and Tables

**Table 1:** Proportion of Imbalanced and Balanced patients reporting certain comorbidities.

Comorbidity	Imbalanced Patients	Balanced Patients	*p*-Value
Osteoporosis	14.4% (*n* = 379/2624)	6.6% (*n* = 482/7292)	<0.001 ^^^
Arthritis	51.6% (*n* = 1361/2638)	31.9% (*n* = 2336/7326)	<0.001 ^^^
Neck Pain	10.8% (*n* = 286/2638)	4.3% (*n* = 318/7326)	<0.001 ^^^
Low Back Pain	54.4% (*n* = 1434/2638)	32.7% (*n* = 2392/7326)	<0.001 ^^^
Depression/Anxiety	1.5% (*n* = 40/2638)	0.6% (*n* = 43/7326)	<0.001 ^^^

Denominators reflect number of patients from each group with valid responses, may not reflect reported total of each group. ^^^ Statistically significant: *p* < 0.05.

**Table 2:** Comparison of median and mean ^(#)^ laboratory values between Imbalanced and Balanced groups.

Lab Value	Imbalanced Patients Intake	Balanced Patients Intake	*p*-Value
Albumin (g/dL) (3.5–5.0) * (IQR)	4.30 (4.00)	4.40 (3.00)	<0.001 ^^^
ALT (U/L) (10–40) * (IQR)	19.00 (11.00)	19.00 (10.00)	<0.001 ^^^
AST (U/L) (5–30) * (IQR)	20.00 (9.00)	21.00 (7.00)	0.002 ^^^
Alkaline phosphatase (U/L) (30–120) * (IQR)	85.00 (35.00)	77.00 (31.00)	0.001 ^^^
Blood urea nitrogen (mg/dL) (8–23) * (IQR)	12.00 (5.00)	12.90 (4.20)	<0.001 ^^^
Calcium, total (mg/dL) (8.2–10.2) * (IQR)	9.30 (0.60)	9.30 (0.50)	0.132
Cholesterol, total (mg/dL) (<200 (desirable)) * (IQR)	200.00 (43.00)	200.00 (49)	0.132
Bicarbonate (mmol/L) (21–28) * (IQR)	23.00 (3.00)	24.00 (3.00)	0.666
Glucose (mg/dL) (70–110) * (IQR)	91.00 (20.00)	88.00 (12.00)	<0.001 ^^^
Iron (µgl/dL) (60–150) * (IQR)	70.00 (37.00)	75 (40.00)	<0.001 ^^^
Phosphorus (mg/dL) (2.3–4.7) * (IQR)	3.40 (0.50)	3.50 (0.70)	0.036 ^^^
Bilirubin, total (mg/dL) (0.3–1.2) * (IQR)	0.40 (0.30)	0.40 (0.10)	0.001 ^^^
Protein, total (g/dL) (6.0–8.0) * (IQR)	7.60 (0.70)	7.60 (0.60)	0.003 ^^^
Triglycerides (mg/dL) (<160) * (IQR)	118.00 (104)	111.00 (89.00)	<0.001 ^^^
Uric acid (mg/dL) (4.0–8.0) * (IQR)	4.50 (1.70)	4.40 (17.70)	0.412
Creatinine (mg/dL) (0.8–1.3) * (IQR)	0.60 (0.20)	0.60 (0.20)	0.037 ^^^
Sodium (mmol/L) (136–142) *^#^ (STD)	139.10 (2.82)	139.22 (2.53)	0.071
Potassium (mmol/L) (3.5–5.0) * (IQR)	4.03 (0.43)	4.03 (0.39)	0.001 ^^^
Chloride (mmol/L) (96–106) *^#^ (STD)	102.51 (3.44)	102.78 (3.01)	0.001 ^^^
Osmolality (mOsm/kg) (275–295) * (IQR)	278.00 (9.00)	278.00 (6.00)	<0.001 ^^^

* Source: AMA Manual of Style [17]. ^^^ Statistically significant: *p* < 0.05. ^#^ Mean value given.

**Table 3:** Comparison of Median (IQR) nutritional intake between Imbalanced and Balanced groups.

Nutritional Parameter	Imbalanced Patients Intake	Balanced Patients Intake	*p*-Value
Energy (kcal) [1600–2200] * (IQR)	1674.00 (942)	1822.00 (1066)	<0.001 ^^^
Dietary Fiber (gm) [25.2–33.6] * (IQR)	12.80 (10.40)	13.60 (10.90)	0.017 ^^^
Protein, total (gm) [46–56] * (IQR)	62.66 (42.39)	69.90 (43.19)	<0.001 ^^^
Thiamine (B1) (mg) [1.1–1.2] * (IQR)	1.29 (0.88)	1.40 (0.95)	0.001 ^^^
B2 (mg) [1–1.3] * (IQR)	1.82 (1.37)	1.88 (1.24)	0.028 ^^^
B3 Niacin (mg) [14–16] * (IQR)	17.46 (13.80)	19.83 (13.36)	<0.001 ^^^
B6 (mg) [1.2–1.5] * (IQR)	1.43 (1.16)	1.53 (1.11)	0.002 ^^^
B12 (mcg) [1.8–2.4] * (IQR)	3.37 (3.83)	3.64 (3.88)	0.048 ^^^
Vitamin D (nmol/L) [50] * (IQR)	55.70 (31.90)	58.10 (29.50)	0.002 ^^^
Vitamin C (mg) [45–90] * (IQR)	54.50 (692.00)	59.60 (99.3)	0.007 ^^^
Ca (mg) [1000–1300] * (IQR)	645.00 (565.00)	659.00 (575.00)	0.166
PO4 (mg) [700–1250] * (IQR)	1044.00 (692.00)	1132.00 (708)	<0.001 ^^^
Alpha carotene (mcg) (IQR)	35.00 (163)	47.00 (224)	0.003 ^^^
Beta Carotene (mcg) [5000–6000] * (IQR)	654 (1655)	740.00 (2018)	0.011 ^^^

* Source: U.S. Department of Health and Human Services and U.S. Department of Agriculture. 2015–2020 Dietary Guidelines for Americans. 8th Edition [26]. ^^^ Statistically significant: *p* < 0.05.

**Table 4:** Comparison of proportion of Imbalanced and Balanced patients reporting difficulty during physical assessment and performance measures.

Functional Test	Imbalanced Patients	Balanced Patients	*p*-Value
Stooping/crouching/kneeling	74.3% (*n* = 1069/1439)	44.7% (*n* = 1307/2927)	<0.001 ^^^
Standing for prolonged periods of time	67.6% (*n* = 968/1433)	36.44% (*n* = 1055/2895)	<0.001 ^^^
Lifting or carrying	52.6% (*n* = 754/1433)	24.3% (*n* = 711/2923)	<0.001 ^^^
Rising from armless chair	48.6% (*n* = 699/1438)	21.0% (*n* = 615/2927)	<0.001 ^^^
Walking up 10 steps	43.8% (*n* = 500/1142)	21.0% (*n* = 569/2711)	<0.001 ^^^
Grasping small objects	32.2% (*n* = 463/1438)	12.8% (*n* = 375/2927)	<0.001 ^^^
Standing up on their own	4.0% (*n* = 65/1628)	0.9% (*n* = 45/4794)	<0.001 ^^^

Denominators reflect number of patients from each group with valid responses, may not reflect reported total of each group. ^^^ Statistically significant: *p* < 0.05.

## Data Availability

Data was gathered from the following website: https://wwwn.cdc.gov/nchs/nhanes/Default.aspx which was accessed on 19 August 2022.
